# Supplementary material for: The Dual Prey-Inactivation Strategy of Spiders—In-Depth Venomic Analysis of Cupiennius salei
Source: Toxins (Basel). 2019 Mar 19;11(3):167. doi: 10.3390/toxins11030167 (PMC6468893; doi:10.3390/toxins11030167)
Supplement: Supplementary file 1 [file toxins-11-00167-s001.zip › Supplementary Fig S1.1-S1.19/Supplementary Fig S1.13.pdf]

Supplementary Fig S1.13

CLUSTAL O(1.2.4) multiple sequence alignment: Phospholipases A2

```
PLA2_CUPSA                     MSSAGWIFVGLLFISPFYEAAQIKDEPSVRKSRQRRSLVDLKDMVKSVTGREGSDFINYG
PLA2_ALOMA                     MSSAGWIFVGLLFISPFYEAAEMKDESSSSRSRQRRSLVDLKDMVKAVTGREGGDFINYG
PLA2_ANCRU                     MSSAGWVFVGLLFISPFYEAAEMKDDSNVIKSRQRRNIMNLRDMVKAVTGREGSDFINYG
PLA2_PHOFE                     MSSAGWIFVGLLFISPFYEATEVKDESTVAKSRQRRNLVNLKDMVKSVTGRDGEDFINYG
TR_A0A087TZ71_STEMI            MSTTGWIFLGILFSSPFIQAAKHKDETTGGTKRHRRSLTDLNSMVQSIIGREGSDFIGYG
GI_XP_015923836.1_PARTE        MSSVYLLIIGLLLFAATNG---DDTQQKDISLRNRRSLTNMYTMWTELTGTEGTKFVNYG
SP_P00630_PA2_APIME            ----MQVVLGSLFLLLLST-------SHGWQIRDRIGDNEL----------EERIIYPGT
                                     :.:* *:                   *.* .  ::          :   :

PLA2_CUPSA                     NWCGLGGSGKP------VDPVDECCQNHDICYDLATSGVCREHGTKGVYQLEYKWRVTDD
PLA2_ALOMA                     NWCGLGGSGKP------VDPVDECCQNHDFCYDLATAGVCREHGNKGVYQLEYKWRVADD
PLA2_ANCRU                     NWCGLGGSGRP------VDPIDECCQNHDICYDLATDGVCEEHGEKGVYQLEYKWKVADD
PLA2_PHOFE                     NWCGLGGSGKP------VDPVDECCQNHDFCYDLATDGVCEEHGEKGVYQLEYKWRVADD
TR_A0A087TZ71_STEMI            NWCGLGGSGRP------VDPIDECCQVHDMCYVLATEGPCKDQGEKGVYQIEYQWSKKSD
GI_XP_015923836.1_PARTE        NYCGLGGSGTP------VDPLDGCCQSHDLCYEEVELSACQQEGEKALYGKNYNYKIEN-
SP_P00630_PA2_APIME            LWCGHGNKSSGPNELGRFKHTDACCRTHDMCPDVMSAGESKHGLTNT-------------
                                :** *...        ..  * **: **:*      . ...   :

PLA2_CUPSA                     GMAVCNDEKDECKMAICFCDAIISKCLKSNIDAYDPKNYHDIDLFQLFKEADYLSS----
PLA2_ALOMA                     GTAVCNDEKDECKMAICFCDAIISNCLKSNIDAYDPKNYHDMDLFQLIQEADFISS----
PLA2_ANCRU                     GTAVCNGEKEPCKMAICFCDAIISNCLKSNIDSYNGQNLHDLDIFQMIKEADWISS----
PLA2_PHOFE                     GTAICNDEKDQCKMAICFCDAIISNCLKSNIDAYNPENFHDLDIFQMIKDADWISS----
TR_A0A087TZ71_STEMI            GTASCKDNADRCP-----------------------------------------------
GI_XP_015923836.1_PARTE        ERATCDKNDDTCATSLCLCDVVLNNCVKSNLHLYNPEYYHQVSISEAMMQGNYLKNIL--
SP_P00630_PA2_APIME            ---------ASHTRLSCDCDDKFYDCLKNSADTISSYFV-GKMYFNLIDTKCYKLEHPVT

PLA2_CUPSA                     -------------------------------
PLA2_ALOMA                     -------------------------------
PLA2_ANCRU                     -------------------------------
PLA2_PHOFE                     -------------------------------
TR_A0A087TZ71_STEMI            -------------------------------
GI_XP_015923836.1_PARTE        -------------------------------
SP_P00630_PA2_APIME            GCGERTEGRCLHYTVDKSKPKVYQWFDLRKY
```

Percent Identity Matrix - created by Clustal2.1

1: PLA2_CUPSA	100.00	88.82	78.82	82.94	62.99	37.35	23.81
2: PLA2_ALOMA	88.82	100.00	81.18	85.29	62.20	36.75	24.60
3: PLA2_ANCRU	78.82	81.18	100.00	85.29	59.84	37.35	23.81
4: PLA2_PHOFE	82.94	85.29	85.29	100.00	61.42	37.95	22.22
5: tr_A0A087TZ71_STEMI	62.99	62.20	59.84	61.42	100.00	39.02	26.19
6: gi_XP_015923836.1_PARTE	37.35	36.75	37.35	37.95	39.02	100.00	15.62
7: SP_P00630_PA2_APIME	23.81	24.60	23.81	22.22	26.19	15.62	100.00

Proteomic coverage trypsin

>PLA2_CUPSA phospholipase A2

MSSAGWIFVG LLFISPFYEA AQIKDEPSVR KSRQRRSLVD LKDMVKSVTG R**EGSDFINYG NWCGLGGSGK PVDPVDECCQ**
NHDICYDLAT SGVCREHGTK **GVYQLEYKWR VTDDGMAVCN DEKDECKMAI CFCDAIISKC LKSNIDAYDP KNYHDIDLFQ**
LFKEADYLSS

Proteomic coverage chymotrypsin

>PLA2_CUPSA phospholipase A2

MSSAGWIFVG LLFISPFYEA AQIKDEPSVR KSRQRRSLVD LKDMVKSVTG R**EGSDFINYG NWCGLGGSGK PVDPVDECCQ**
NHDICYDLAT S**GVCREHGTK GVY**QLEY**KWR VTDDGMAVCN DEKDECKMAI CFCDAIISKC L**KSNIDAYDP KNYHDIDLFQ
LFKEADYLSS

Supplementary Fig S1.13 Multiple sequence alignment of PLA2 from the venom glands of *C. salei* (PLA2_CUPSA [MH766636]), *A. marikovskyi* (PLA2_ALOMA), *Ancylometes rufus* (PLA2_ANCRU]), *S. mimosarum* (tr_A0A087TZ71_STEMI), *P. tepidariorum* (gi_XP_015923836.1_PARTE), and the honeybee *Apis mellifera* (sp_P00630_PA2_APIME). Signal peptides are marked in black characters, cysteines in white with a black box, positively charged amino acids in pink, negatively charged amino acids in blue, hydrophobic amino acids in red and polar amino acids in green. Metal binding residues are highlighted in gray and catalytic residues in yellow. The propeptide from *A. mellifera* is highlighted in green and a theoretically cutting site for spider PLA2 are boxed with red and white characters.
